# Typhus Fever

**Published:** 1899-04-29

**Authors:** 


### TYPHUS FEVER.

In a special report on a case of typhus fever by Dr. Waldo, medical officer of health to the parish of St. George the Martyr, the infection is very carefully traced back from case to case; so far back, indeed, that Dr. Waldo is able to show that the whole series sprang from an individual who, alone out of his family, escaped isolation and disinfection in a previous outbreak. On February 20th, 1899, the case in question was notified from a house in Southwark Bridge Road, and on inquiry it was found that several of her relatives had been attacked with mysterious illnesses, and on investigation the following sequence of cases was made out. In October, 1898, a case of typhus fever, the origin of which could not be ascertained, occurred in the parish. The case was removed to hospital, the house was closed for disinfection, and Dr. Waldo induced all the inmates with the exception of the father to enter the Reception House which had been opened for the purpose of sheltering people during the process of disinfection. The father, however, went to Bermondsey, and slept two nights at a house in Minto Street, which we will call A. In this house, A, there were resident four families, and on December 18th Mrs. F., a resident in this house, was admitted to Guy's Hospital, where she was treated for supposed enteric fever, and was discharged to a convalescent home on January 22nd. On December 24th the son and daughter-in-law of the man who had taken the infection from Southwark to Minto Street left A, the house to which the infection had been brought by the father, and removed to a house in Tabard Street, Newington, which we will call B. Here the son was taken ill on January 13th, 1899, and was removed in a delirious condition to the Newington Workhouse, where he died on January 18th. The wife of the last patient was taken ill on about the same date, and died at B on January 14th. Returning now to A, we find that Mrs. F, who was first attacked there on the night before she went into Guy's, called at another house in Minto Street, C, and arranged for one of her boys being taken there as a lodger. At the same time she interviewed a woman from another house, D, who undertook to take charge of a second son. Towards the end of January this latter son was taken ill and removed to St. Olave's Union Infirmary, where he still remained at the time the report was presented. Now going back to the house C, in Minto Street, we find it inhabited by the W. family — father, mother, three sons, and two daughters, and the recently-admitted son of Mrs. F. Seven of these eight persons were admitted to hospital at various dates up to March 7th, and all these cases have been recognised either at the time or subsequently as typhus fever. Two of these patients had died at the time of the report. Now we come to the case reported to Dr. Waldo on February 20th, 1899, from the Southwark Bridge Road. She, it turned out, had removed to that address only two days previously, having come from Weston Street, Bermondsey, and on inquiry it also came out that she had sat up one night with her brother Richard W., the head of the above-mentioned W. family, while a patient at King's College Hospital, where he died on January 30th. We understand that, since this report was sent in, another member of the W. family has been removed from the house in Weston Street, the case being notified as suffering from typhus fever. The above history is a good example of painstaking work in tracing out infection, and it serves well to show how virulent a disease typhus becomes when it finds a fitting soil. Dr. Waldo very reasonably concludes that the infection was carried out of the parish by the father of the first patient, and that had he entered the Reception House along with the rest of the family the centre of infection from which all this mischief arose would have been entirely stamped out.

